# Biosynthesis of Sandalwood Oil: *Santalum album* CYP76F Cytochromes P450 Produce Santalols and Bergamotol

**DOI:** 10.1371/journal.pone.0075053

**Published:** 2013-09-18

**Authors:** Maria L. Diaz-Chavez, Jessie Moniodis, Lufiani L. Madilao, Sharon Jancsik, Christopher I. Keeling, Elizabeth L. Barbour, Emilio L. Ghisalberti, Julie A. Plummer, Christopher G. Jones, Jörg Bohlmann

**Affiliations:** 1 Michael Smith Laboratories, University of British Columbia, Vancouver, British Columbia, Canada; 2 School of Plant Biology, University of Western Australia, Crawley, Western Australia, Australia; 3 School of Chemistry and Biochemistry, University of Western Australia, Crawley, Western Australia, Australia; University of Houston, United States of America

## Abstract

Sandalwood oil is one of the world’s most highly prized essential oils, appearing in many high-end perfumes and fragrances. Extracted from the mature heartwood of several *Santalum* species, sandalwood oil is comprised mainly of sesquiterpene olefins and alcohols. Four sesquiterpenols, α-, β-, and *epi*-β-santalol and α-*exo-*bergamotol, make up approximately 90% of the oil of *Santalum album.* These compounds are the hydroxylated analogues of α-, β-, and *epi*-β-santalene and α-*exo*-bergamotene. By mining a transcriptome database of *S. album* for candidate cytochrome P450 genes, we cloned and characterized cDNAs encoding a small family of ten cytochrome P450-dependent monooxygenases annotated as *Sa*CYP76F37v1, *Sa*CYP76F37v2, *Sa*CYP76F38v1, *Sa*CYP76F38v2, *Sa*CYP76F39v1, *Sa*CYP76F39v2, *Sa*CYP76F40, *Sa*CYP76F41, *Sa*CYP76F42, and *Sa*CYP76F43. Nine of these genes were functionally characterized using *in vitro* assays and yeast *in vivo* assays to encode santalene/bergamotene oxidases and bergamotene oxidases. These results provide a foundation for production of sandalwood oil for the fragrance industry by means of metabolic engineering, as demonstrated with proof-of-concept formation of santalols and bergamotol in engineered yeast cells, simultaneously addressing conservation challenges by reducing pressure on supply of sandalwood from native forests.

## Introduction

**Figure 1:**
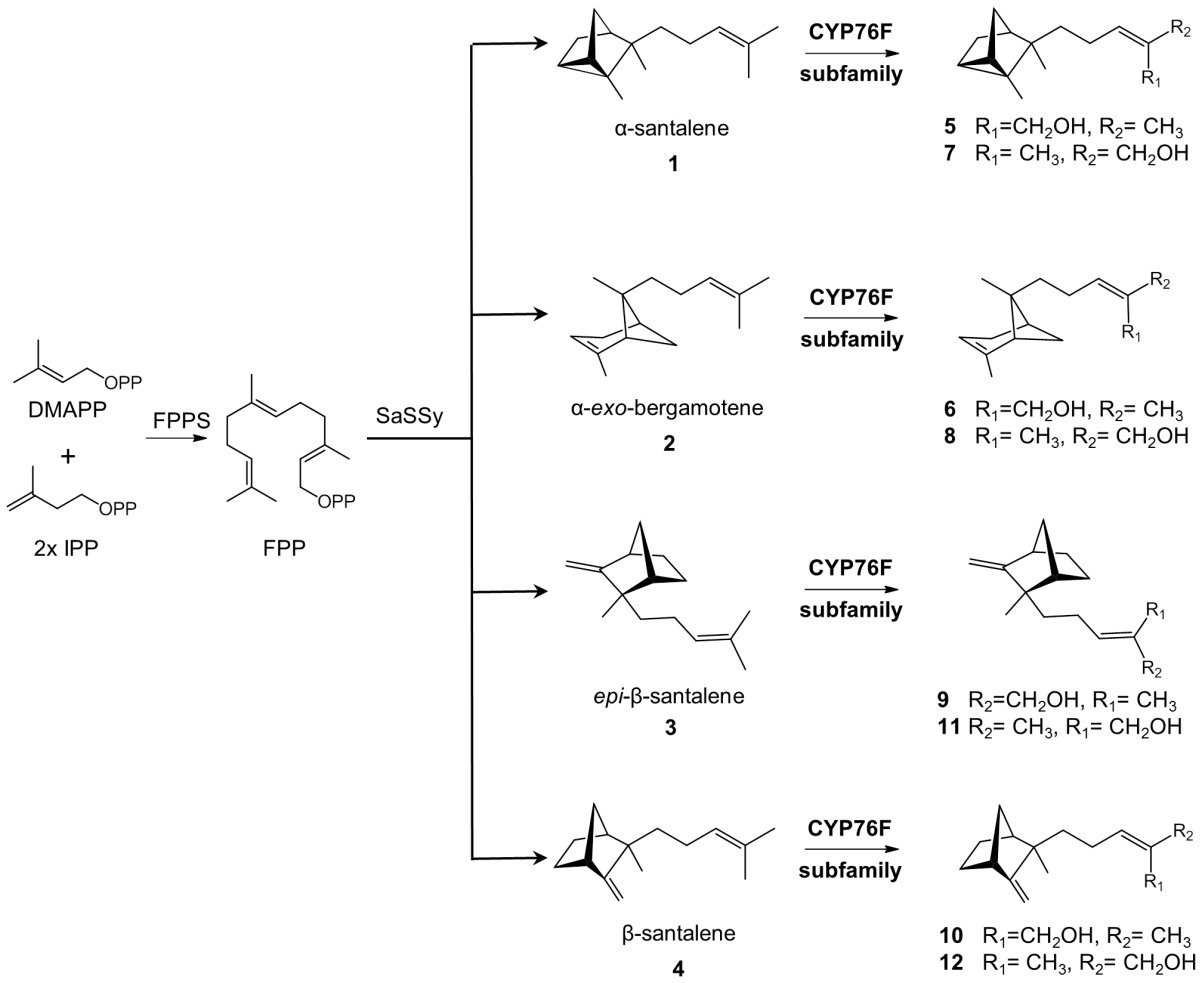
Schematic biosynthetic pathway for santalols and bergamotol in sandalwood. Compounds identified with numbers are: α-santalene (1), α-*exo*-bergamotene (2), *epi*-β-santalene (3), β-santalene (4), (*Z*)-α-santalol (5), (*E*)-α-santalol (7), (*Z*)-α-*exo*-bergamotol (6), (*E*)-α-*exo*-bergamotol (8), (*Z*)-*epi*-β-santalol (9), (*E*)-*epi*-β-santalol (11), (*Z*)-β-santalol (10), (*E*)-β-santalol (12). Numbers match the numbers in Table 1. DMADP, dimethylallyl diphosphate; IPP, isopentenyl diphosphate; FPP, farnesyl diphosphate; FPPS, farnesyl diphosphate synthase; *Sa*SSy, *S. album* santalene synthase.

Sandalwood is the general name for woody perennials of the *Santalum* genus (Santalaceae), which are exploited for their fragrant heartwood. Sandalwoods are slow growing hemiparasitic trees distributed throughout the tropical and temperate regions of India, Indonesia, Australia and the Pacific Islands [1],[2]. The oil extracted from the stems and roots are highly sought after by the fragrance and perfume industry. *Santalum album*, also known as tropical or Indian sandalwood, is the most valuable of the commercially used species due to the high heartwood oil content (6–10% by dry weight) and desirable odor characteristics. Approximately 90% of *S. album* essential oil is composed of the sesquiterpene alcohols α-, β-, and *epi*-β-santalol and α-*exo-*bergamotol (Figure 1). The α- and β-santalols are the most important contributors to sandalwood oil fragrance [3]–[5]. Lanceol and α-bisabolol are also found in modest concentrations [6]. While the demand for sandalwood oil is increasing, disease, grazing animals and unsustainable exploitation of sandalwood trees has led to the demise of many natural populations. Plantations provide a more sustainable alternative to wild harvesting; however, slow growth rates, high potential for disease and substantial variation in oil yield hamper productivity. Alternatively, chemical approaches to synthesize the santalols have been attempted [7]–[9], but multiple low-recovery steps make chemical synthesis uneconomical at an industrial scale.

Investigations into alternative, more sustainable strategies to produce sandalwood oil include improved plantation systems through development of predictive marker systems for oil biosynthesis in developing heartwood of the slow growing trees, and metabolic engineering of heterologous production systems. Key to these approaches is the elucidation of the biosynthesis of the santalols, bergamotols, and other sesquiterpene compounds characteristic of sandalwood oil. The first step in santalol and bergamotol biosynthesis is the generation of farnesyl diphosphate (FPP) from dimethylallyl diphosphate and isoprenyl diphosphate, catalyzed by FPP synthase (FPPS). FPP is cyclized by santalene synthase (*Sa*SSy), a previously characterized sesquiterpene synthase [10], which produces a mixture of santalenes (α-, β- and *epi*-β-santalene) and α-*exo*-bergamotene. Since *Sa*SSy generated four structurally similar products, it seemed plausible that a single, multi-substrate cytochrome P450 dependent monooxygenase (P450) could oxidize α-, β-, *epi*-β-santalene and bergamotene to produce α-, β-, *epi*-β-santalols and bergamotol, respectively (Figure 1). Alternatively, different cytochromes P450 could be involved in the oxidation of the different santalenes and bergamotene.

Here, we describe the discovery, cloning and functional characterization of a family of ten *S. album* P450s of the new CYP76F subfamily and an NADPH-dependent cytochrome P450 reductase (CPR) involved in santalol/bergamotol biosynthesis.

## Results

### Gene Discovery and Full-Length (FL)cDNA Cloning

A *S. album* trancriptome assembly of 31,461 isotigs was blastx searched for candidate CPRs and P450s potentially involved in the hydroxylation of santalenes and bergamotene. Two *Sa*CPRs were identified using *Arabidopsis thaliana* CPRs (CAB58575.1, CAB58576.1) as search sequences. FLcDNAs *Sa*CPR1 and *Sa*CPR2 were 70% identical and 82% similar at the amino acid level. Searches for P450s were performed with a set of known plant P450s of the CYP71, CYP72 and CYP76 families, which include P450s with known functions in terpenoid biosynthesis [11]–[13].

**Figure 2:**
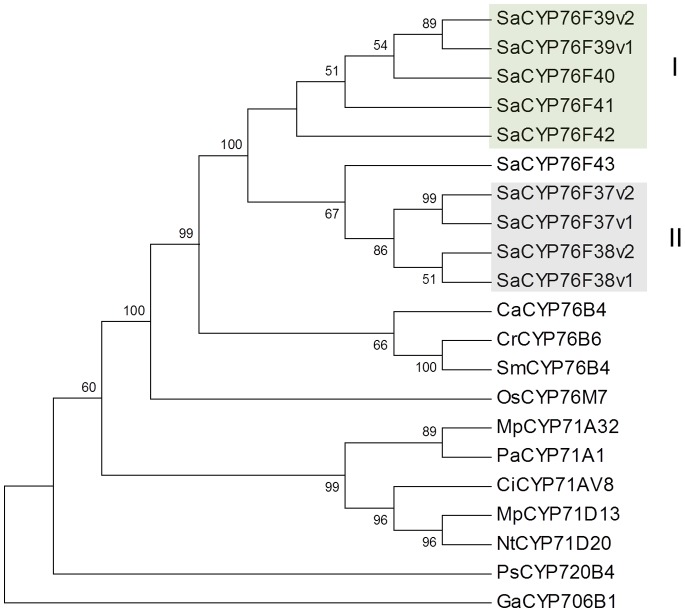
Phylogenetic tree of *S. album* CYP76F proteins and related terpene-modifying P450s. The neighbor-joining tree was constructed with members of the CYP71 clan, using *Picea sitchensis Ps*CYP720B4 (ADR78276) as an outgroup. *S. album* CYP76F proteins fell into two clades, clade I santalene/bergamotene oxidases and clade II bergamotene oxidases. *Ca*CYP76B4, *Camptotheca acuminata* putative geraniol-10-hydroxylase (AES93118); *Cr*CYP76B6, *Catharanthus roseus* geraniol 10-hydroxylase (Q8VWZ7); *Sm*CYP76B4, *Swertia mussotii* geraniol 10-hydroxylase (D1MI46); *Os*CYP76M7 *Oryza sativa ent*-cassadiene C11a-hydroxylase (NP_001047185); *Mp*CYP71A32, *Mentha x piperita* menthofuran synthase (Q947B7); *Pa*CYP71A1, *Persea americana* (P24465); *Ci*CYP71AV8, *Cichorium intybus* valencene oxidase (ADM86719); *Mp*CYP71D13, *Mentha x piperita*; (−)-limonene-3-hydroxylase (AY281027); *Nt*CYP71D20, *Nicotiana tabacum*, 5-*epi*-aristolochene-1,3-dihydroxylase (AF368376); *Ga*CYP706B1, *Gossypium arboreum* (+)-delta-cadinene-8-hydroxylase (AAK60517). This work: *Sa*CYP76F37v1 (KC533717); *Sa*CYP76F37v2 (KC698966); *Sa*CYP76F38v1 (KC533715); *Sa*CYP76F38v2 (KC533718); *Sa*CYP76F39v1 (KC533716); *Sa*CYP76F39v2 (KC698967); *Sa*CYP76F40 (KC698968); *Sa*CYP76F41 (KC698969); *Sa*CYP76F42 (KC698965); *Sa*CYP76F43 (KC533719).

Transcripts of the CYP76 family were among the most abundant P450s in the *S. album* transcriptome and assembled into two different isogroups and two individual isotigs (Table S1). Isogroup 1 consisted of 2,143 reads including 1,107 unique reads assembled into three isotigs. It generated a consensus sequence of 1,917 base pairs and an open reading frame (ORF) of 1,530 bp. Isogroup 2 consisted of 228 reads including 140 unique reads assembled into two isotigs. Both isotigs share a consensus ORF of 1,530 bp. A separate isotig consisted of 11 reads generating a partial sequence of 1,200 bp. Another separate isotig contained one partial sequence of 277 bp with several stop codons. Isogroups 1 and 2 were selected for FLcDNA cloning. PCR amplification with primers designed according to isogroup 1 resulted in a single unique FLcDNA clone designated as *Sa*CYP76F38v1. PCR amplification with primers based on isogroup 2 resulted in nine different cDNAs clones designated as *Sa*CYP76F37v1, *Sa*CYP76F37v2, *Sa*CYP76F38v1, *Sa*CYP76F38v2, *Sa*CYP76F39v1, *Sa*CYP76F39v2, *Sa*CYP76F40, *Sa*CYP76F41, *Sa*CYP76F42, and *Sa*CYP76F43. The predicted CYP76F proteins were 94–99% identical to each other and contained motifs characteristic of eukaryotic P450s including a proline-rich region near the N-terminal membrane-anchoring domain, the oxygen-binding domain and the highly conserved heme binding motif (Figure S1). A blastp search of the deduced amino acid sequences against the NCBI GenBank protein database identified best matches to a putative P450 from *Vitis vinifera* (XP_002281735) with 62–64% identity, and CYP76B6 geraniol hydroxylase (CAC80883) from *Catharanthus roseus* [14] with 53–54% identity. A phylogeny with related plant P450s (Figure 2) showed the *S. album* CYP76F proteins form two separate clades, I and II, and are closest to the CYP76B cluster of other species.

### Expression of Recombinant *Sa*CYP76Fs in Yeast

*Sa*CYP76F FLcDNAs were expressed together with *Sa*CPR2 in yeast cells, and microsomes were isolated for *in vitro* P450 enzyme assays. Microsome preparations for all ten *Sa*CYP76Fs, except *Sa*CYP76F43, displayed characteristic P450 CO difference spectra. The P450 content of the microsomal preparations ranged from 0.2 to 1.6 µM (Figure S2).

### In Vitro Functional Identification of Clade I *Sa*CYP76Fs using a Blend of Sesquiterpenes

Microsome preparations were screened for sesquiterpene oxidase activity using NADPH and a defined sesquiterpene mixture of α-, β- and *epi*-β-santalene and α-*exo*-bergamotene as substrate. These sesquiterpenes are not commercially available and were produced by expression of *Sa*SSy in yeast (Figure S3A). Product formation was measured by gas chromatography mass spectrometry (GCMS).

**Figure 3:**
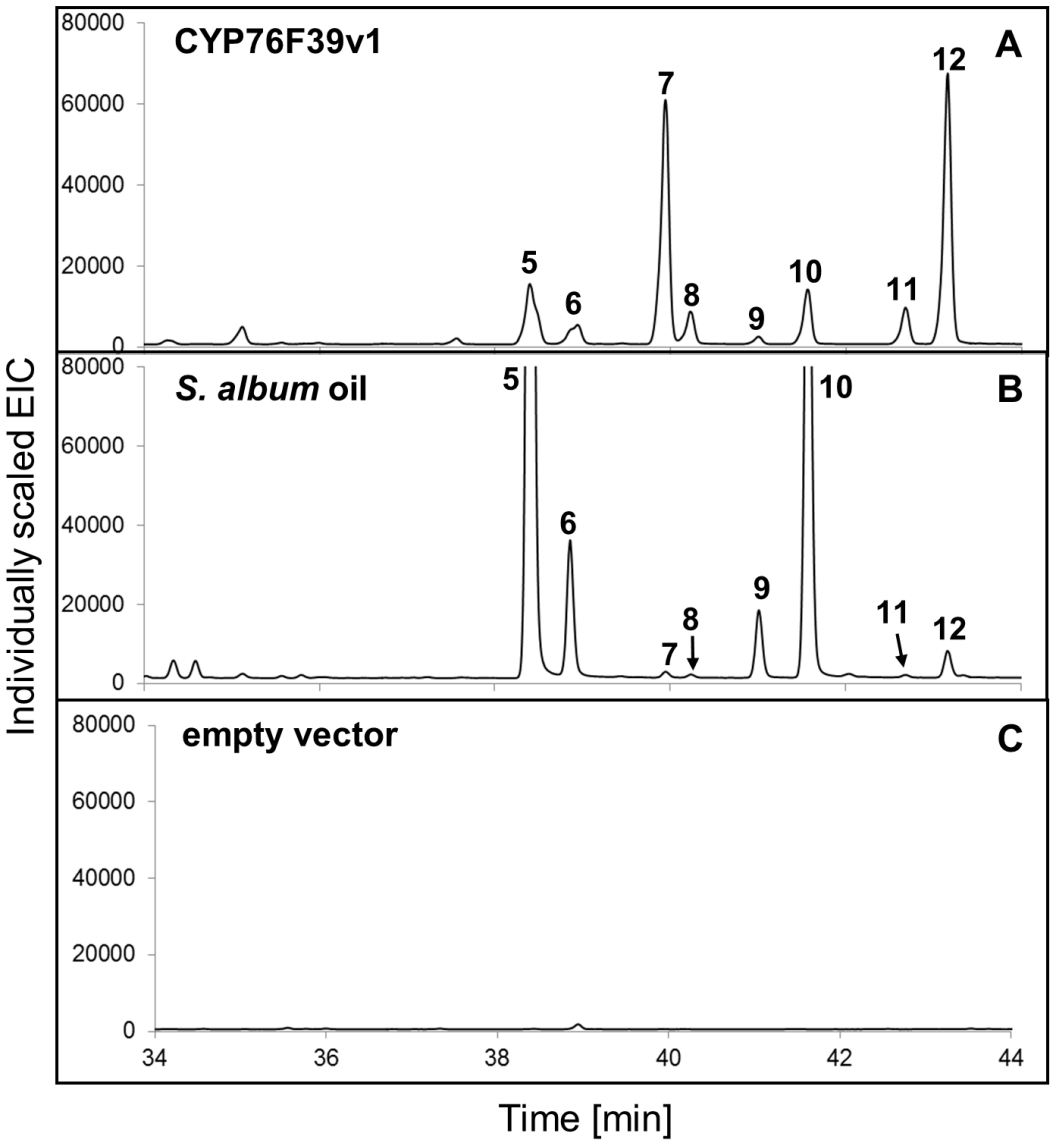
GCMS analysis of products formed *in vitro* with *Sa*CYP76F39v1. A sesquiterpene mixture of α-, β- and *epi*-β-santalene and α-*exo*-bergamotene (Figure S3) was incubated with microsomes containing *Sa*CYP76F39v1 and *Sa*CPR produced in yeast. (**A**) Product profile (extracted ion chromatogram, EIC) of assays with *Sa*CYP76F39v1. (**B**) Authentic *S. album* oil. (**C**) Control assays were performed with microsomes isolated from yeast cells transformed with the empty vector. Mass spectra of compounds corresponding to peaks 5**–**12 identified in assays with *Sa*CYP76F39v1 (left panel) and *S. album* oil (right panel) are shown in Figure S4. Peak numbers match the numbers in Table 1 and Figure 1.

Focusing initially on the clade I *Sa*CYP76F39v1, we found that microsomes containing this P450 catalyzed the hydroxylation of the three santalenes and α-*exo*-bergamotene, leading to eight different compounds identified as (*Z*)*-* and (*E*)-α-santalol (5 and 7), (*Z*)*-* and (*E*)-α-*exo*-bergamotol (6 and 8), (*Z*)*-* and (*E*)-*epi*-β-santalol (9 and 11) and (*Z*)*-* and (*E*)-β-santalol (10 and 12) (Figure 3A and Table 1; peak numbers in Figures match the numbers in Table 1). Products were identified based on matches of their mass spectra (Figure S4) with entries in the NIST and Wiley libraries and by matches of retention indices obtained on two different GC matrices (Table 1) with retention indices reported in the literature [15],[16]. In addition, comparison of the product profile with the profile of an authentic sandalwood oil sample (Figure 3B) showed identical retention times and nearly identical mass spectra (Figure S4) for all eight compounds that were present both in the product of the enzyme assay and in the oil, albeit in different proportions. No product formation was detected in the absence of NADPH or with microsomes from yeast carrying an empty vector (Figure 3C).

**Table 1:** Retention indices of sesquiterpenes and sesquiterpenols identified in the enzyme assays with cytochromes P450 of the *S. album* CYP76F subfamily and of sesquiterpene alcohols of *S. album* oil.

No^1^	Compound	LRI^2^ DBwax	LRI^3^ HP5
1	α-santalene	1579	1423
2	α-*exo*-bergamotene	1592	1437
3	*epi*-β-santalene	1637	1450
4	β-santalene	1652	1463
5	(*Z*)-α-santalol	2343	1676
6	(*Z*)-α-*exo*-bergamotol	2353	1692
7	(*E*)-α-santalol	2382	1697
8	(*E*)-α-*exo*-bergamotol	2389	1711
9	(*Z*)-*ep*i-β-santalol	2409	1703
10	(*Z*)-β-santalol	2423	1717
11	(*E*)-*epi*-β-santalol (tentative)	2452	1726
12	(*E*)-β-santalol	2465	1738

1 These numbers are used as identifiers for compounds and corresponding peaks in.

GC traces throughout the paper and figures.

2 Linear retention indices (LRI) measured on a DBwax column.

3 Linear retention indices (LRI) measured on an HP5 column.

The sesquiterpenol profile produced *in vitro* by microsomes containing *Sa*CYP76F39v1 matched the overall sesquiterpenol composition of *S. album* oil; however, the relative amounts of individual stereoisomers differed (Figure 3). CYP76F39v1 produced (*E*)-α-santalol (7) and (*Z*)-α-santalol (5) in a ratio of approximately 5∶1, and (*E*)-β-santalol (12) and (*Z*)-β-santalol (10) in a ratio of approximately 4∶1, while (*Z*)-α-santalol (5) and (*Z*)-β-santalol (10) are the more dominant isomers in the oil.

**Figure 4:**
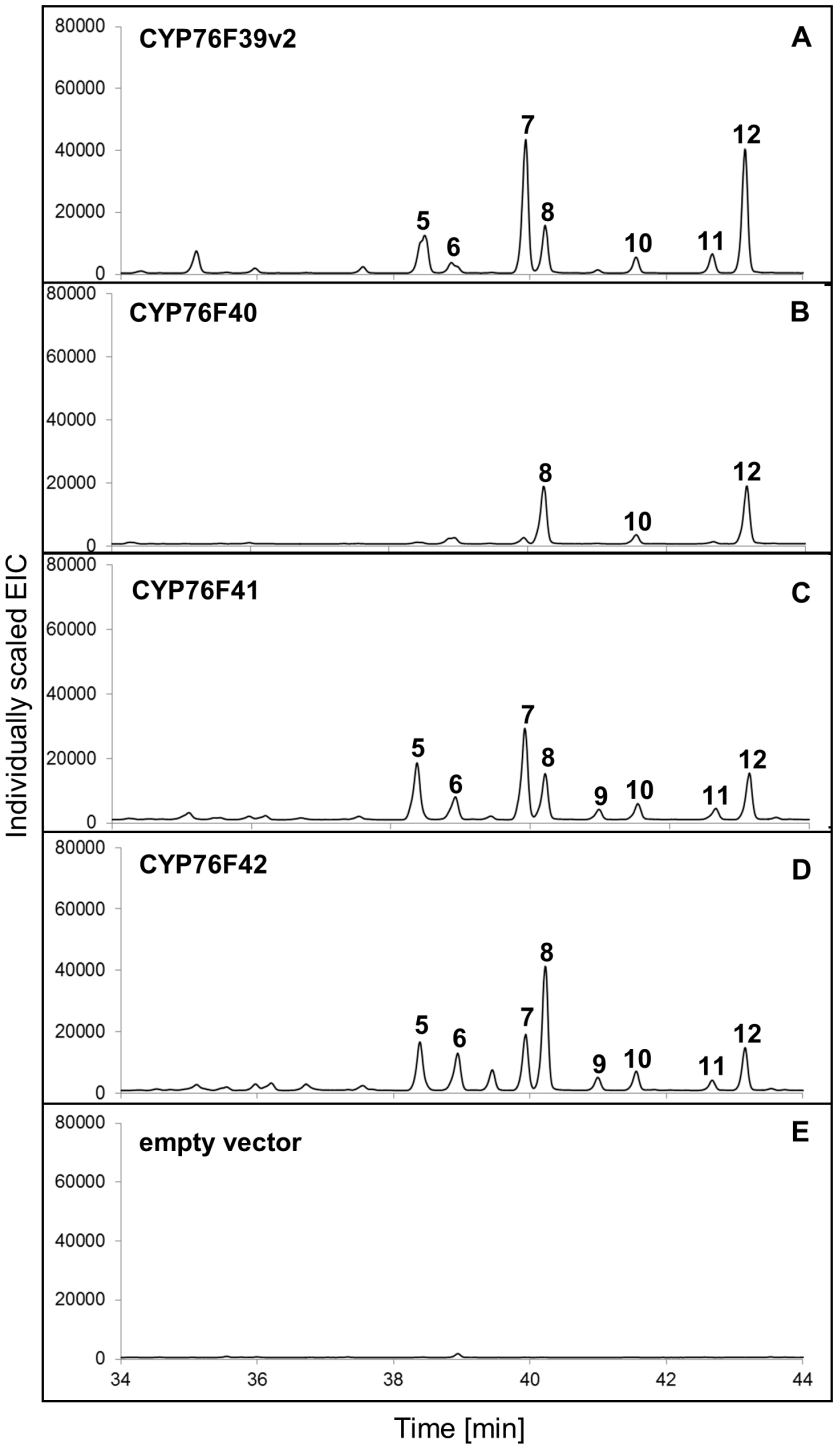
GCMS analysis of products formed *in vitro* with clade I *Sa*CYP76Fs. GCMS analysis (extracted ion chromatograms) of products formed *in vitro* with (**A**) *Sa*CYP76F39v2; (**B**) *Sa*CYP76F40; (**C**) *Sa*CYP76F41; (**D**) *Sa*CYP76F42. Assays were performed with a sesquiterpene mixture of α-, β- and *epi*-β-santalene and α-*exo*-bergamotene (Figure S3) as substrate and microsomes prepared from yeast transformed with *Sa*CPR, individual clade I candidate *Sa*CYP76F cDNAs, or (**E**) empty vector as control. Peak numbers match the numbers in Table 1 and Figure 1.

Other clade I P450s, specifically *Sa*CYP76F39v2, *Sa*CYP76F40, *Sa*CYP76F41, and *Sa*CYP76F42, gave product profiles similar to that observed with CYP76F39v1 (Figure 4 A–D). The major products formed by microsomes containing *Sa*CYP76F40 or *Sa*CYP76F42 were (*E*)- α-*exo*-bergamotol (8) and (*E*)-β-santalol (12).

### In vitro Functional Identification of Clade II SaCYP76Fs using a Blend of Sesquiterpenes

**Figure 5:**
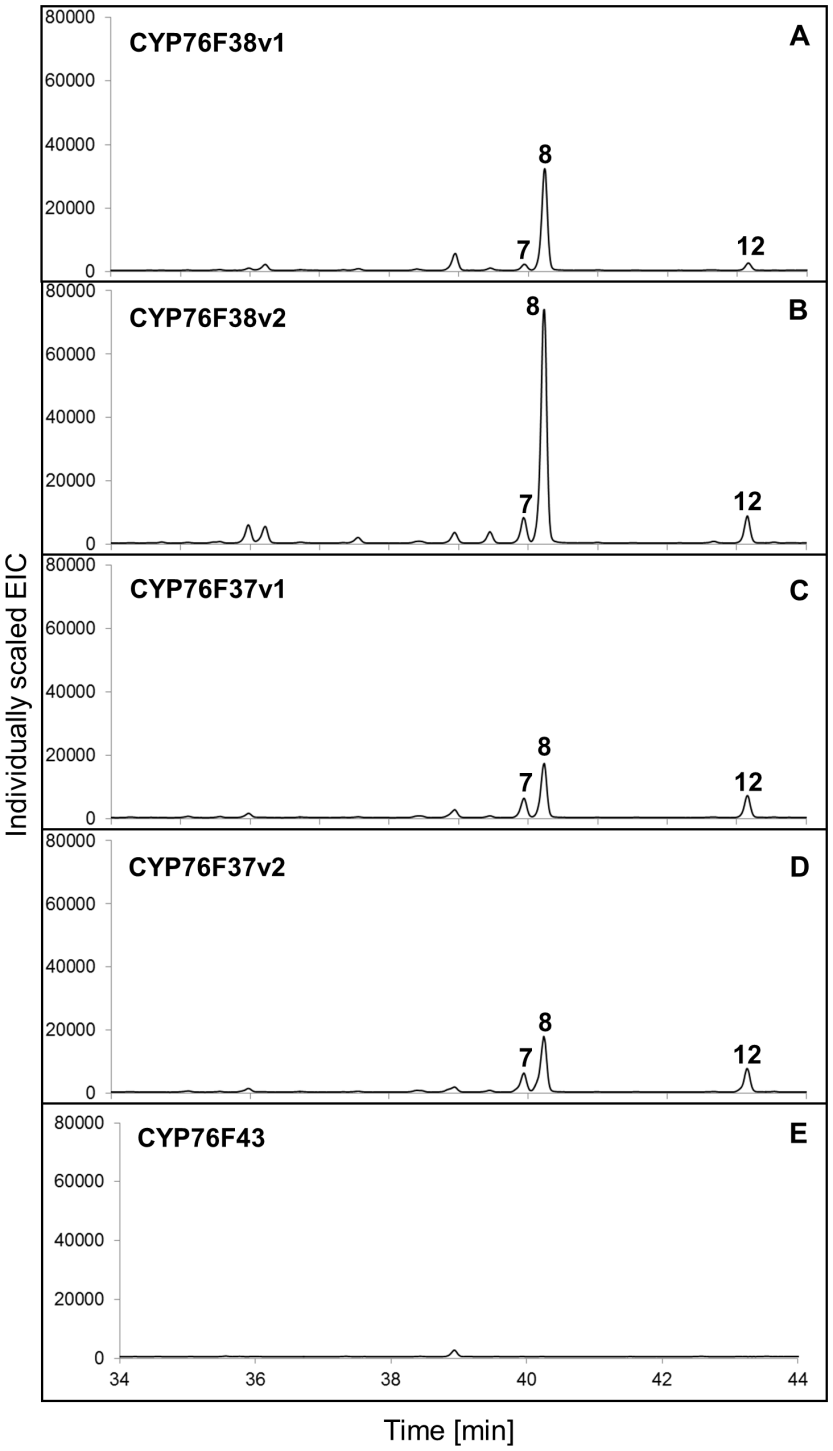
GCMS analysis of products formed *in vitro* with clade II *Sa*CYP76Fs. GCMS analysis (extracted ion chromatograms) of products formed *in vitro* with (**A**) *Sa*CYP76F38v1; (**B**) *Sa*CYP76F38v2; (**C**) *Sa*CYP76F37v1; (**D**) *Sa*CYP76F37v2. Assays were performed with a sesquiterpene mixture of α-, β- and *epi*-β-santalene and α-*exo*-bergamotene (Figure S3) as substrate and microsomes prepared from yeast transformed with *Sa*CPR, individual clade II candidate *Sa*CYP76F cDNAs, or (**E**) empty vector as control. Peak numbers match the numbers in Table 1 and Figure 1.

In contrast to the clade I *Sa*CYP76Fs, which each gave the same eight sesquiterpenol products, microsomes containing clade II members *Sa*CYP76F37v1, *Sa*CYP76F37v2, *Sa*CYP76F38v1, and *Sa*CYP76F38v2 gave only three products identified as (*E*)-α-*exo*-bergamotol (8) as the major product and (*E*)-α-santalol (7) and (*E*)-β-santalol (12) as minor products (Figure 5A–D). No activity was found with *Sa*CYP76F43 (Figure 5E) possibly due to low expression in yeast as evidenced by the corresponding CO difference spectrum (Figure S2).

### Characterization of Clade I and Clade II SaCYP76Fs using Individual Sesquiterpenes

Although the authentic candidate substrates are not available in pure form, we could partially separate the sesquiterpenes of the mixture of α-, β- and *epi*-β-santalene and α-*exo*-bergamotene (Figure S3). Three different fractions containing mainly α-santalene (1) (Figure S3B), α-*exo*-bergamotene (2) (Figure S3C), or *epi*-β-santalene (3) and β-santalene (4) (Figure S3D) were used as individual substrates in assays with microsomes containing *Sa*CYP76F39v1, representing clade I, or *Sa*CYP76F37v1, representing clade II. *Sa*CYP76F39v1 with α-santalene produced both (*Z*)- and (*E*)-α-santalol (5 and 7; Figure S5A), while only (*E*)-α-santalol (7) formation was detected with *Sa*CYP76F37v1 (Figure S5D). With α-*exo*-bergamotene, *Sa*CYP76F39v1 produced (*Z*)- and (*E*)-α-*exo*-bergamotol (6 and 8; Figure S5B), while only (*E*)-α-*exo*-bergamotol (8) formation was detected with *Sa*CYP76F37v1 (Figure S5E). *Sa*CYP76F39v1 gave four products, (*Z*)- and (*E*)-*epi*-β-santalol (9 and 11) and (*Z*)- and (*E*)-β-santalol (10 and 12), in assays with *epi*-β-santalene and β-santalene (Figure S5C), whereas only (*E*)-β-santalol (12) was detected in assays with *Sa*CYP76F37v1 (Figure S5F). These results confirmed the activities seen with microsome *in vitro* assays with the mixture of santalenes and bergamotene.

### Substrate Specificity and Kinetic Properties of SaCYP76Fs

**Figure 6:**
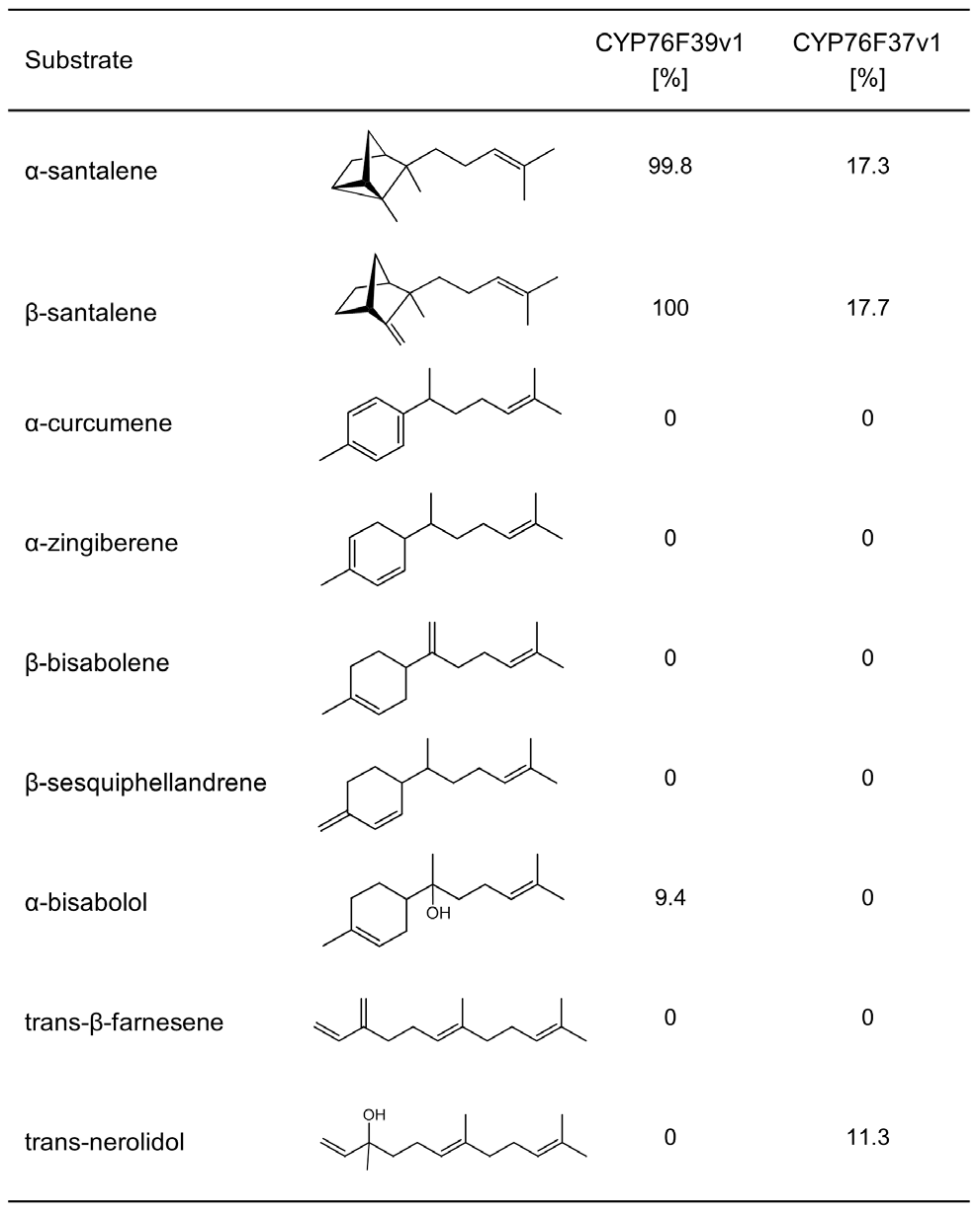
Relative activities of *Sa*CYP76F39v1 and *Sa*CYP76F37v1 with different sesquiterpenes. Relative activities represent rate of product formation relative to product formation by *Sa*CYP76F39v1 with β-santalene.

To test the range of substrates potentially converted by the clade I and clade II *Sa*CYP76F enzymes, we assayed *Sa*CYP76F37v1 and *Sa*CYP76F39v1with a set of sesquiterpenes which resemble santalenes in the acyclic isoprenyl side chain (Figure 6). Of the nine different substrates tested, *Sa*CYP76F39v1 efficiently converted only the two santalenes, while it showed low activity with α-bisabolol and was not active with α-curcumene, zingiberene, β-bisabolene, β-sesquiphellandrene, farnesene, and trans-nerolidol. These results demonstrated a narrow substrate selectivity of *Sa*CYP76F39v1 with sesquiterpenes relevant for sandalwood oil biosynthesis. Similarly, *Sa*CYP76F37v1 was selectively active with the two santalenes and trans-nerolidol.

Apparent K_m_ values of *Sa*CYP76F39v1 and *Sa*CYP76F37v1 were, respectively, 25.92 (±0.11) µM and 133 (±0.41) µM with α-santalene, and respectively, 34.82 (±0.41) µM and 157 (±0.17) µM with β-santalene. The k_cat_ values obtained for *Sa*CYP76F39v1 were 1.12 s^−1^ with α-santalene and 1.17 s^−1^ with β-santalene. The k_cat_/K_m_ values for *Sa*CYP76F39v1 were 4.3×10^4^ s^−1^ M^−1^ with α-santalene and 3.3×10^4^ s^−1^ M^−1^ with β-santalene. The k_cat_ values obtained for *Sa*CYP76F37v1 were 0.2 s^−1^ with α-santalene and 0.13 s^−1^ with β-santalene. The k_cat_/K_m_ values for *Sa*CYP76F37v1 were 1.5×10^3^ s^−1^ M^−1^ with α-santalene and 8.1×10^2^ s^−1^ M^−1^ with β-santalene.

### Formation of Santalols and Bergamotol in Transformed Yeast Cells

**Figure 7:**
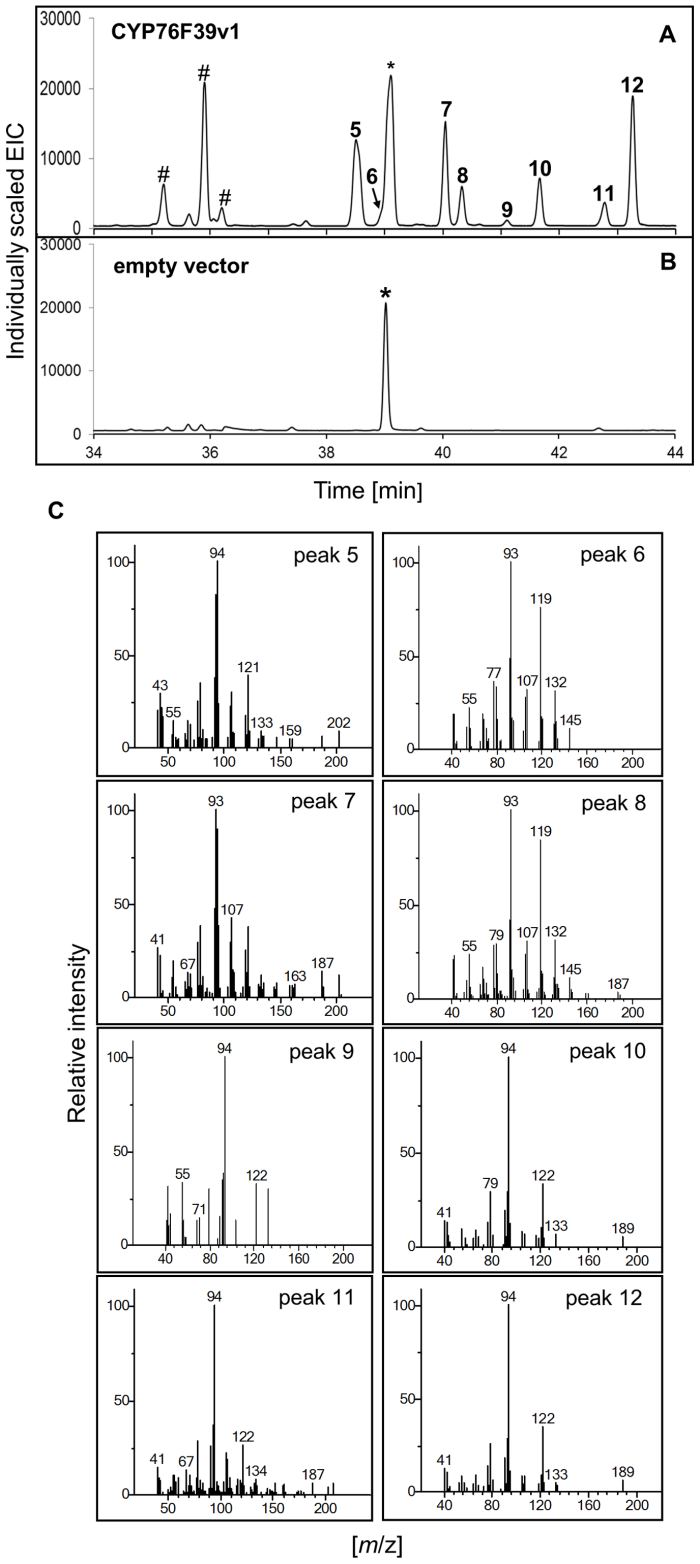
GCMS analysis of products formed *in vivo* with *Sa*CYP76F39v1. GCMS analysis (extracted ion chromatograms) of compounds formed *in vivo* in yeast cells expressing *Sa*SSY, *Sa*CPR2 and (**A**) *Sa*CYP76F39v1 or (**B**) an empty vector. (**C**) Mass spectra of compounds corresponding to peaks 5–12 identified in (**A**). Peak numbers match the numbers in Table 1 and Figure 1. Peaks in (**A**) and (**B**) marked with symbol (*) correspond to farnesol also produced in yeast cells without *Sa*CYP76F. Peaks in (**A**) marked with symbol (#) represent yeast *in vivo* modifications of santalols (see Figure S6).

To test the potential for using *Sa*CYP76F cDNAs to produce santalols and bergamotol *in vivo*, we first expressed the previously characterized *Sa*SSy and *Sa*FPPS cDNAs [10] in yeast to form the known *Sa*SSy products α-santalene (1), α-*exo*-bergamotene (2), *epi*-β-santalene (3) and β-santalene (4). These four sesquiterpenes were detected in transformed yeast cells (Figure S6), but were not released with detectable amounts into the culture medium. No differences were observed between cells expressing *Sa*SSy with or without the additional *Sa*FPPS suggesting that endogenous yeast FPP is accessible for *Sa*SSy to produce santalenes and bergamotene. We then tested product formation with the additional expression of *Sa*CPR2 and *Sa*CYP76F candidate cDNAs. GCMS analysis of yeast cells expressing *Sa*CYP76F39v1, *Sa*CPR2 and *Sa*SSy showed a product profile of eight sesquiterpenols identified as (*Z*)- and (*E*)-α-santalol (5 and 7), (*Z*)- and (*E*)-α-*exo*-bergamotol (6 and 8), (*Z*)- and (*E*)-*epi*-β-santalene (9 and 11) and (*Z*)- and (*E*)-β-santalene (10 and 12) (Figure 7A), similar to the product profile of the *in vitro* assays (Figure 3A). The product peak for (*Z*)-α-*exo*-bergamotol (6) overlapped with a peak corresponding to (*E*,*E*)-farnesol, which was produced in yeast independent of the *Sa*CYP76F39v1 (Figure 7B).

Apparently, a fraction of the sesquiterpenol produced by recombinant yeast expressing *Sa*SSy, *Sa*CPR2 and *Sa*CYP76F39v1 were modified to unknown compounds (identified with hash marks in Figure 6A). When untransformed yeast cells were incubated with authentic sandalwood oil, we found the same unknown compounds (Figure S7), implying that these compounds are not direct products of *Sa*CYP76F39v1, but are produced by an endogenous activity of yeast converting sandalwood sesquiterpenols.

*In vivo* analysis of the other *Sa*CYP76F clade I members gave product profiles with nearly identical ratios (Figure S8) as observed with the corresponding *in vitro* assays with the microsomal preparations (Figure 4). Yeast cells expressing clade II *Sa*CYP76Fs produced mostly (*E*)-α-*exo*-bergamotol (8) similar to the products formed in the *in vitro* assays, but only traces of santalols (7 and 12) (Figure S9). Again, no activity was found with CYP76F43.

### Effect of CPR1 and CPR2

To test if substituting *Sa*CPR1 and *Sa*CPR2, which are 70% identical at the protein level, could affect changes in product profiles, we tested both CPRs in yeast *in vivo* experiments with representative class I and class II *Sa*CYP76F, CYP76F39v1 and CYP76F38v1. No differences were observed in the products and their relative abundances.

## Discussion

Using transcriptome analysis, cloning and functional characterization of recombinant P450s, we identified a new CYP76F subfamily in *S. album* involved in the biosynthesis of α-, β- and *epi*-β-santalols and bergamotols. The different *Sa*CYP76Fs catalyze hydroxylations of santalenes and/or bergamotene products of *Sa*SSy at the terminal allylic methyl groups. Clade I *Sa*CYP76F enzymes produced both (*Z*) and (*E*) stereoisomers of α-, β- and *epi*-β-santalols and bergamotols. The P450 product ratios of (*Z*) and (*E*) stereoisomers of α- and β-santalol were approximately 1∶5 and 1∶4, respectively, while the oil harvested from the mature heartwood of *S. album* trees contained mainly the (*Z*) alcohols [17],[18]. There are several possible explanations for the difference in the ratio of stereoisomers found in the enzyme product profile and in the oil extracted from trees. Importantly, we excluded the possibility that the activity of *Sa*CYP76Fs was non-specific towards a range of different substrates, since only products of *Sa*SSy were preferred substrates when compared with other similar sesquiterpenes. However, it is important to note that conditions of yeast cells and *in vitro* assays are different compared to the physiological conditions *in planta*, which might explain the differences of product stereoisomers observed. It is possible that subtle changes in the shape and size of the active site under different conditions might result in the olefin precursors being oxidized in different configurations. It is also important to note that the products detected in *in vitro* microsome assays and in yeast *in vivo* assays were formed and accumulated over a period of minutes to hours. In contrast, the oil extracted from mature heartwood is the product of biosynthesis and accumulation that occurs over a much longer time period of many years. Isomerization, perhaps catalyzed by an isomerase, may be possible in the trees, however may not have been mimicked with the conditions of the *in vitro* or yeast *in vivo* enzyme assays used here. Although the ten P450s isolated in this work are the most abundant P450s in the sandalwood transcriptome sequences, it is also possible that additional sandalwood P450s exist that are similarly active on the santalenes and bergamotene substrates, but generating predominantly the (*Z*) stereoisomer. We will be exploring this possibility with further screening of the *S. album* P450 family.

The CYP76 gene family is part of the CYP71 clan, which includes P450 families involved in plant primary and secondary metabolism. Previously functionally characterized CYP76 members are involved in xenobiotic detoxification [19], oxidation of iridoid monoterpenoids [14],[20], and oxidation of diterpenes [21],[22]. The CYP76F members described here for sesquiterpene hydroxylation add a new dimension to the known functional space of the CYP76 family. The number of CYP76 genes is highly variable in different plant species. For example, papaya (*Carica papaya*) contains three CYP76 genes, *A. thaliana* has nine CYP76 genes, and grapevine (*Vitis vinifera*) has 24 CYP76 genes [11],[23]. The ten *S. album* CYP76F members described here were identified based on transcriptome sequencing and may not represent the full complement of CYP76 genes of this species. In the absence of a genome sequence of *S. album*, it is not clear if any of these genes represent pairs of allelic variants. The *S. album* CYP76F members separate into two clades, clade I and II. Although there is overlap in their product profiles, clade I members formed preferentially santalols, whereas clade II members produced preferentially (*E*)-α-*exo*-bergamotol.

The CYP76 and CPR cDNAs described here, combined with previously cloned santalene synthases [10], provide a biotechnology opportunity to produce valuable components of sandalwood oil. Our initial results demonstrate the potential of transformed yeast cells for production of santalols and bergamotols. As a proof-of-concept, we reconstructed the pathways for biosynthesis of santalols and bergamotols in yeast cells using the multi-product *Sa*SSy and *Sa*CPR in combination with different multi-substrate *Sa*CYP76Fs. These results provide a foundation for further metabolic engineering to improve yields and target product specificities.

The cloned terpene synthases [10],[24] and P450s (this study) of sandalwood oil biosynthesis can also be explored as biomarkers to monitor the onset of oil formation in sandalwood plantations or for the development of genetic markers for tree improvement. In this context, it is important to note that very little is known about the cell types and the molecular events that control spatial and temporal patterns of the onset of biosynthesis of sandalwood oil. In fact, the spatial and temporal patterns of the onset of sandalwood oil biosynthesis are not well known, beyond the association of oil accumulation in the aging heartwood of sandalwood stems and roots. The aging heartwood of sandalwood trees provides an extremely difficult system to study with biochemical tools. Thus, the genes described here and in previous work [10] and their possible applications for metabolic engineering of sandalwood oil biosynthesis and the development of molecular markers are likely to become more important as worldwide demand for sandalwood products increase and as natural resources of *S. album* continue to decline.

## Materials and Methods

### Materials

The *Saccharomyces cerevisiae* yeast strain used in this study was BY4741 (MATa his3Δ1 leu2Δ0 met15Δ0 ura3Δ0). *Escherichia coli* α-Select Chemically Competent Cells (Bioline) were used for routine cloning and plasmid propagation. The sesquiterpene olefins α-, β- and *epi*-β-santalene, and α-*exo*-bergamotene are not commercially available, but can be produced by expression of *Sa*SSy in yeast [10]. A sesquiterpene oil containing α-, β- and *epi*-β-santalene, and α-*exo*-bergamotene was produced in an industrial scale fermentation system by Allylix, Inc. (Kentucky, USA). The mixture was separated using silver nitrate impregnated TLC plates according to Daramwar *et al*. [25]; fractions were scraped from TLC plates and sesquiterpenes eluted with pentane followed by GCMS analysis for purity. Other sesquiterpenes, specifically bisabolol, trans-β-farnesene and trans-nerolidol were purchased from SIGMA. Zingiberine, α-curcumene, β-bisabolene and β-sesquiphellandrene were from our in house collection of sesquiterpene standards isolated from natural sources.

### Transcriptome Sequences

A cDNA library made from *Santalum album* xylem was sequenced with Sanger technologies generating 11,520 paired end sequences [10]. 454 Titanium sequencing of the cDNA library generated an additional 902,111 sequence reads. The transcriptome assembly was done using both the 454 and Sanger sequences with Roche Newbler assembler version 2.6 under default parameters, which generated a total of 31,461 isotigs.

### Cloning of P450 and CPR FLcDNAs and Yeast Transformation

FLcDNAs were amplified by PCR using Phusion Hot Start II DNA Polymerase (Thermo Scientific) with gene specific primers (Table S2) and cDNA prepared from *S. album* wood cores and leaves as template. PCR conditions included initial denaturing at 98°C for 3 min, two cycles at 98°C for 10 sec, Tm-2°C for 20 sec, and 72°C for 30 sec, followed by 30 cycles at 98°C for 10 sec, Tm for 20 sec and 72°C for 30 sec, and termination for 7 min at 72°C. PCR products were gel purified and cloned into the pJET1.2 vector (Fermentas). Constructs designated pJET1.2-*Sa*CYP76F37 through pJET1.2-*Sa*CYP76F43, pJET1.2-*Sa*CPR1 and pJET1.2-*Sa*CPR2 were sequence verified. *Sa*CYP76F FLcDNAs were subcloned into yeast expression vector pYEDP60 following the User Cloning method [26]. *Sa*SSY (HQ343276) and *Sa*FPPS (HQ343283) cDNAs [10] were cloned, respectively, into the NotI-Bgl II and BamHI-XhoI sites of the dual expression vector pESC-LEU2d by In-Fusion Cloning (Clontech). *Sa*CPR1 and *Sa*CPR2 were cloned individually into the EcoRI-NotI sites of the dual expression vector pESC-HIS (Stratagene). Plasmid transformation of yeast strain BY4741 was done using the LiCl method Gietz *et al*. [27]. Transformed yeast strains were selected on plates with appropriate synthetic complete drop-out selection medium and grown at 30°C for 48 h.

### Microsome Preparation

For microsome isolation, BY4741 cells were transformed with plasmids harboring P450 or CPR. Microsome membranes were prepared from 250 ml cultures according to Pompom *et al*. [28]. In brief, a 5 ml overnight culture was used to inoculate 50 ml of SD-selective media starting at an OD_600_ of 0.2 and grown at 30°C, 170 rpm for 24 h. A volume of 200 ml YPDE medium (1% yeast extract, 2% bacto-peptone, 5% ethanol, 2% dextrose) was inoculated with the 50 ml culture and incubated for another 24 h at 30°C, 170 rpm. Cells were collected by centrifugation for 10 min at 1,000×*g* and induced with 2% galactose in 250 ml YP medium at 30°C, 170 rpm for 12–16 h. Yeast cells were pelleted by centrifugation at 2,000×*g* for 10 min, washed once with 5 ml TEK (50 mM Tris-HCl pH 7.5, 1 mM EDTA, 100 mM KCl) and suspended in TES2 buffer (50 mM Tris-HCl pH 7.5, 1 mM EDTA, 600 mM sorbitol, 5 mM DTT and 0.25 mM PMSF). All subsequent steps were performed at 4°C. Yeast cell were disrupted mechanically using acid-washed glass beads (425–600 µm, Sigma) and vigorous manual shaking for 3×30 sec. The cell homogenate was centrifuged at 10,000×*g* for 15 min followed by ultracentrifugation of the supernatant at 100,000×*g* for 1 h. Microsomes were suspended and homogenized in a buffer containing 50 mM Tris-HCl buffer pH 7.5, 1 mM EDTA and 30% (v/v) glycerol, and used directly for enzyme assays or stored at −80°C.

### CPR Activity and P450 CO Spectra

Activity of recombinant *Sa*CPRs was assayed using the Cytochrome C Reductase (NADPH) assay kit (Sigma). CO difference spectra of recombinant P450s were measured according to Guengerich *et al*. [29].

### In Vitro P450 Assays

Microsome preparations containing candidate P450 and CPR were assayed for their capacity to oxidize sesquiterpenes. The reaction mixtures contained 50 mM potassium phosphate pH 7.5, 0.8 mM NADPH and 40 µM of substrate in a total volume of 400 µl. Enzyme reactions were initiated by adding 50 µl of the microsome preparation, incubated at 30°C for 2 h with shaking and stopped by adding 500 µl of hexane. The organic layer was transferred to a new GC vial and concentrated under N_2_ gas to about 100 µl followed by GCMS analysis. For kinetic analysis, enzyme assays were performed as above with the following modifications: Assays were performed in a total volume of 400 µl with either 17 pmol of *Sa*CYP7639v1 protein or 35 pmol of *Sa*CYP7637v1 protein, and substrate concentrations of 12 to 138 µM of α-santalene or β-santalene; assays were incubated for 20 min.

### Yeast Metabolic Engineering

To assess the production of santalols/bergamotol in a yeast system, the yeast strain BY4741 was co-transformed with plasmids containing cDNAs for *Sa*FPPS, *Sa*SSY, *Sa*CPR, and a candidate CYP76F. Recombinant yeast was initially grown overnight at 30°C in 5 ml of 2% dextrose in minimal selective media. The next day, a 50 ml culture was initiated at a starting OD_600_ of 0.2 and grown at 30°C with shaking at 170 rpm until the culture reached an OD_600_ of 0.6–0.8. Expression was initiated by transfer into minimal selective media with 2% galactose and grown for 14–16 h. Yeast cells were harvested by centrifugation at 1,000×*g* for 10 min and washed once with 5 ml sterile ddH_2_O. Cells were extracted twice by vortexing for 1 min with 2 ml hexane and 250 µl acid-washed glass beads (425–600 µm, Sigma). Pooled extracts were transferred to a clean test-tube containing anhydrous Na_2_SO_4_ and evaporated under a gentle stream of N_2_ gas to about 200 µl. The samples were transferred to a GC glass vial for GCMS analysis or stored at −80°C.

### GCMS Analysis

GCMS analysis was carried out on an Agilent 7890A/5975C GCMS system operating in electron ionization selected ion monitoring (SIM)-scan mode. Samples were analyzed on both an HP5 (non-polar; 30 m×0.25 mm ID×0.25 µm thickness) and a DB-Wax fused silica column (polar; 30 m×0.25 mm ID×0.25 µm thickness). In both cases, the injector was operated in pulsed splitless mode with the injector temperature maintained at 250°C. Helium was used as the carrier gas with a flow rate of 0.8 ml min^−1^ and pulsed pressure set at 25 psi for 0.5 min. Scan range: m/z 40–500; SIM: m/z 93, 94, 105, 107, 119, 122 and 202 [dwell time 50 msec]. The oven program for the HP5 column was: 40°C for 3 min; ramp of 10°C min^−1^ to 130°C, 2°C min^−1^ to 180°C, 50°C min^−1^ to 300°C; 300°C for 10 min. The oven program for the DB-wax column was: 40°C for 3 min; ramp of 10°C min^−1^ to 130°C, 2°C min^−1^ to 200°C, 50°C min^−1^ to 250°C; 250°C for 15 min. Chemstation software was used for data acquisition and processing. Compounds were identified by comparison of mass spectral with authentic standards and the NIST/EPA/NIH mass spectral library v2.0 and by comparison of retention indices with those appearing in other publications [15],[16].

### Phylogenetic Analysis

Phylogenetic analysis was performed using the software MEGA version 4 [30] employing the neighbor-joining (NJ) algorithm with default parameters. Bootstrap (500 replications) confidence values over 50% are displayed at branch points.

### Accession Numbers

The cDNA sequences described in this paper have been submitted to GenBankTM/EBI with accession numbers: *Sa*CYP76F37v1 (KC533717); *Sa*CYP76F37v2 (KC698966); *Sa*CYP76F38v1 (KC533715); *Sa*CYP76F38v2 (KC533718); *Sa*CYP76F39v1 (KC533716); *Sa*CYP76F39v2 (KC698967); *Sa*CYP76F40 (KC698968); *Sa*CYP76F41 (KC698969); *Sa*CYP76F42 (KC698965); *Sa*CYP76F43 (KC533719); SaCPR1 (KC842187); SaCPR2 (KC842188).

## Supporting Information

Figure S1 **Amino acid sequence alignment of** ***S. album*** **CYP76F genes.** *Sa*CYP76F37v1, *Sa*CYP76F37v2, *Sa*CYP76F38v1, *Sa*CYP76F38v2, *Sa*CYP76F39v1, *Sa*CYP76F39v2, *Sa*CYP76F40, *Sa*CYP76F41, *Sa*CYP76F42 and *Sa*CYP76F43. Red, dark grey and light grey shading denote 100% and 80% and 50% conserved residues, respectively. Horizontal arrows denote the proline region (a), O_2_ binding motif (b) and heme binding motif (c). Boxes indicate the putative substrate recognition sites (SRS) regions originally described by Gotoh [31]. Multiple sequence alignment was performed with the software CLUSTALW [32] and visualized with Gendoc v2.7.
(TIF)

Figure S2 **Reduced CO-difference spectra of isolated microsomes containing** ***S. album*** **CYP76F proteins.** CO difference spectra of microsomal fractions from *S. cerevisiae* harboring a cytochrome P450 or an empty vector are shown. Concentration of *Sa*CYP76F proteins are given based on an extinction coefficient of 91,000 M^−1^cm^−1^.
(TIF)

Figure S3 **GCMS analysis (extracted ion chromatogram) of a sesquiterpene mixture and fractions separated by TLC.** The sesquiterpene mixture was produced with a recombinant yeast strain expressing *Sa*SSy (10) and was provide to us by Allylix Inc. It contained (**A**) α-santalene (1), α-*exo*-bergamotene (2**)**, *epi*-β-santalene (3**)**, β-santalene (4). The mixture was separated by TLC into three fractions containing mainly (**B**) α-santalene (1**)**; (**C**) α-*exo*-bergamotene (2**)**; or (**D**) β-santalene (4**)**. Mass spectra of peaks 1 to 4 are provided. Peak numbers match the numbers in Table 1 and Figure 1.
(TIF)

Figure S4 **Mass spectra of products formed** ***in vitro*** **with** ***Sa*** **CYP76F39v1.** Mass spectra of compounds corresponding to peaks 5 - 12 shown in Figure 3 and identified in assays with CYP76F39v1 (left panel) and *S. album* oil (right panel). Peak numbers match the numbers in Table 1, Figure 1, and Figure 3.
(JPG)

Figure S5 **GCMS analysis (extracted ion chromatogram) of products formed** ***in vitro*** **with** ***Sa*** **CYP76F39v1 or** ***Sa*** **CYP76F37v1 using partially purified substrates.** Product profile in assays with *Sa*CYP76F39v1 using (**A**) α-santalene, (**B**) α-*exo*-bergamotene, or (**C**) *epi*-β-santalene and β-santalene as substrate. Product profile in assays with *Sa*CYP76F37v1 using (**D**) α-santalene, (**E**) α-*exo*-bergamotene, or (**F**) *epi*-β-santalene and β-santalene as substrate. (**G**) Products were identified by comparison to authentic standards. Peak numbers match the numbers in Table 1 and Figure 1.
(TIF)

Figure S6 **GCMS analysis (extracted ion chromatogram) and mass spectra of sesquiterpenes produced in yeast expressing** ***Sa*** **SSy.** (**A**) GCMS analysis of sesquiterpenes extracted from pelleted yeast cells expressing *Sa*SSy. (**B**) Mass spectra of peaks 1–4: α-santalene (**1)**, α-*exo*-bergamotene (**2)**, *epi*-β-santalene (**3)**, and β-santalene (**4)**. Compounds were identified by comparison to an authentic standard and retention indices. Peak numbers match the numbers in Table 1 and Figure 1.
(TIF)

Figure S7 **Modification of sandalwood oil compounds in yeast cell culture.** GCMS analysis of sesquiterpenols of natural sandalwood oil sample before (**A**) and after (**B**) overnight incubation with yeast cells, which do not contain a *Sa*CYP76F gene. Peaks in (**B**) marked with symbol (#) represent yeast *in vivo* modifications of santalols independent of *Sa*CYP76F. Peak numbers match the numbers in Table 1 and Fig. 1.
(TIF)

Figure S8 **GCMS analysis of products formed** ***in vivo*** **with clade I** ***Sa*** **CYP76Fs.** GCMS analysis (extracted ion chromatograms) of compounds formed *in vivo* in yeast cells expressing *Sa*SSy, *Sa*CPR2 and (**A**) *Sa*CYP76F39v2, (**B**) *Sa*CYP76F40, (**C**) *Sa*CYP76F41, or (**D**) *Sa*CYP76F42. Peak numbers match the numbers in Table 1 and Figure 1. Peaks marked with symbol (*) correspond to farnesol produced also in yeast cells without *Sa*CYP76F. Peaks in marked with symbol (#) represent yeast *in vivo* modifications of santalols (see Figure S7).
(TIF)

Figure S9 **GCMS analysis of products formed** ***in vivo*** **with clade II** ***Sa*** **CYP76Fs.** GCMS analysis (extracted ion chromatograms) of compounds formed in yeast cells expressing *Sa*SSy, *Sa*CPR2 and (**A**) *Sa*CYP76F38v1, (**B**) *Sa*CYP76F38v2, (**C**) *Sa*CYP76F37v1, (**D**) *Sa*CYP76F37v2, or (**E**) *Sa*CYP76F43. Peak numbers match the numbers in Table 1 and Figure 1. Peaks marked with symbol (*) correspond to farnesol produced also in yeast cells without *Sa*CYP76F. Peaks in marked with symbol (#) represent yeast *in vivo* modifications of santalols (see Figure S7).
(TIF)

Table S1 Summary of transcriptome mining for CYP76 family members in the *S. album* Sanger and 454 sequence data.
(DOCX)

Table S2 Primers designed for amplification of cDNAs from *S. album*.
(DOCX)
