# Supplementary figures and images for: Biosynthesis of Sandalwood Oil: Santalum album CYP76F Cytochromes P450 Produce Santalols and Bergamotol
Source: PLoS One. 2013 Sep 18;8(9):e75053. doi: 10.1371/journal.pone.0075053 (PMC3854609; doi:10.1371/journal.pone.0075053)

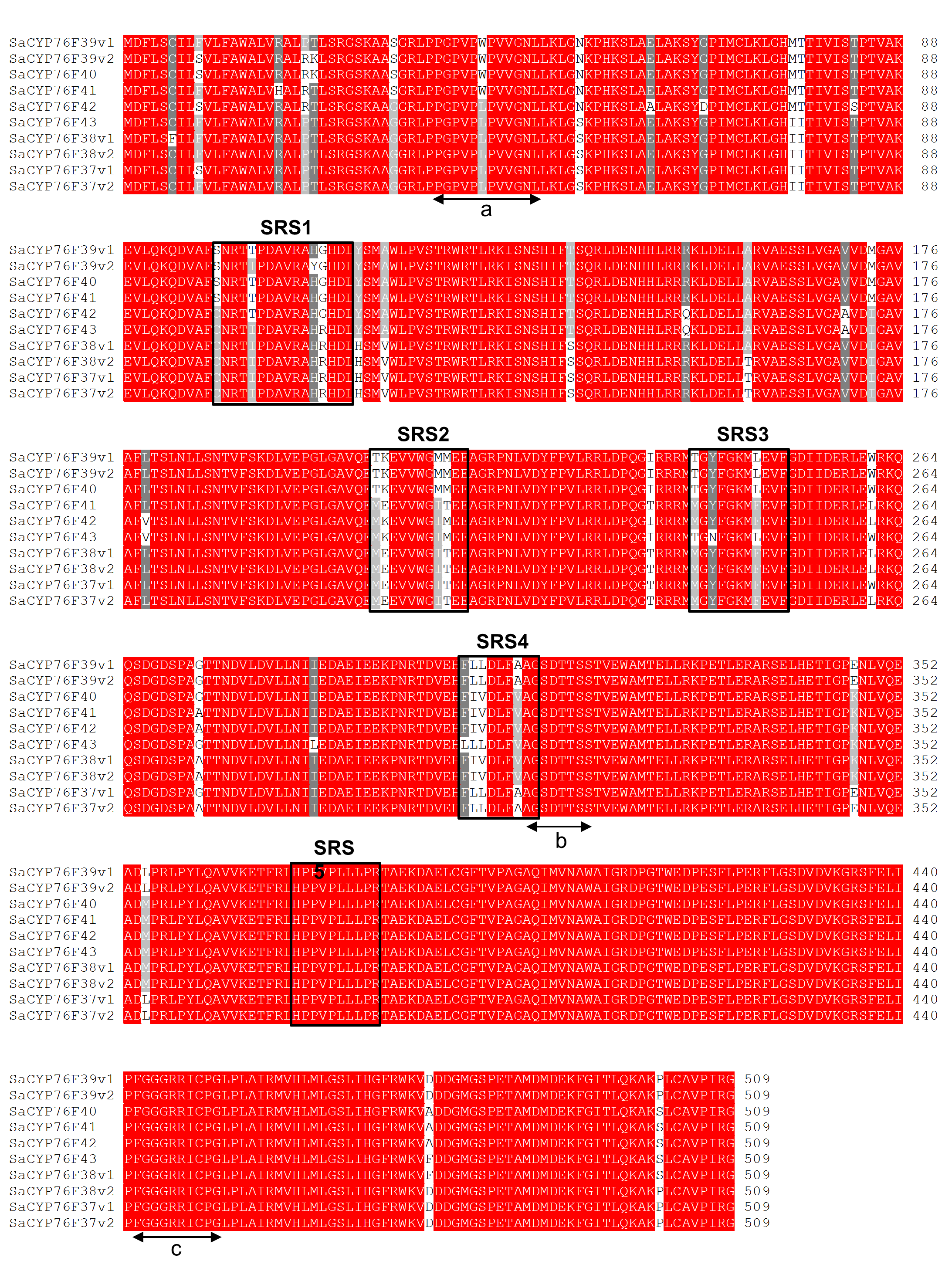

Supplement: Figure S1 — Amino acid sequence alignment of S. album CYP76F genes. SaCYP76F37v1, SaCYP76F37v2, SaCYP76F38v1, SaCYP76F38v2, SaCYP76F39v1, SaCYP76F39v2, SaCYP76F40, SaCYP76F41, SaCYP76F42 and SaCYP76F43. Red, dark grey and light grey shading denote 100% and 80% and 50% conserved residues, respectively. Horizontal arrows denote the proline region (a), O2 binding motif (b) and heme binding motif (c). Boxes indicate the putative substrate recognition sites (SRS) regions originally described by Gotoh [31]. Multiple sequence alignment was performed with the software CLUSTALW [32] and visualized with Gendoc v2.7. (TIF) [file pone.0075053.s001.tif]

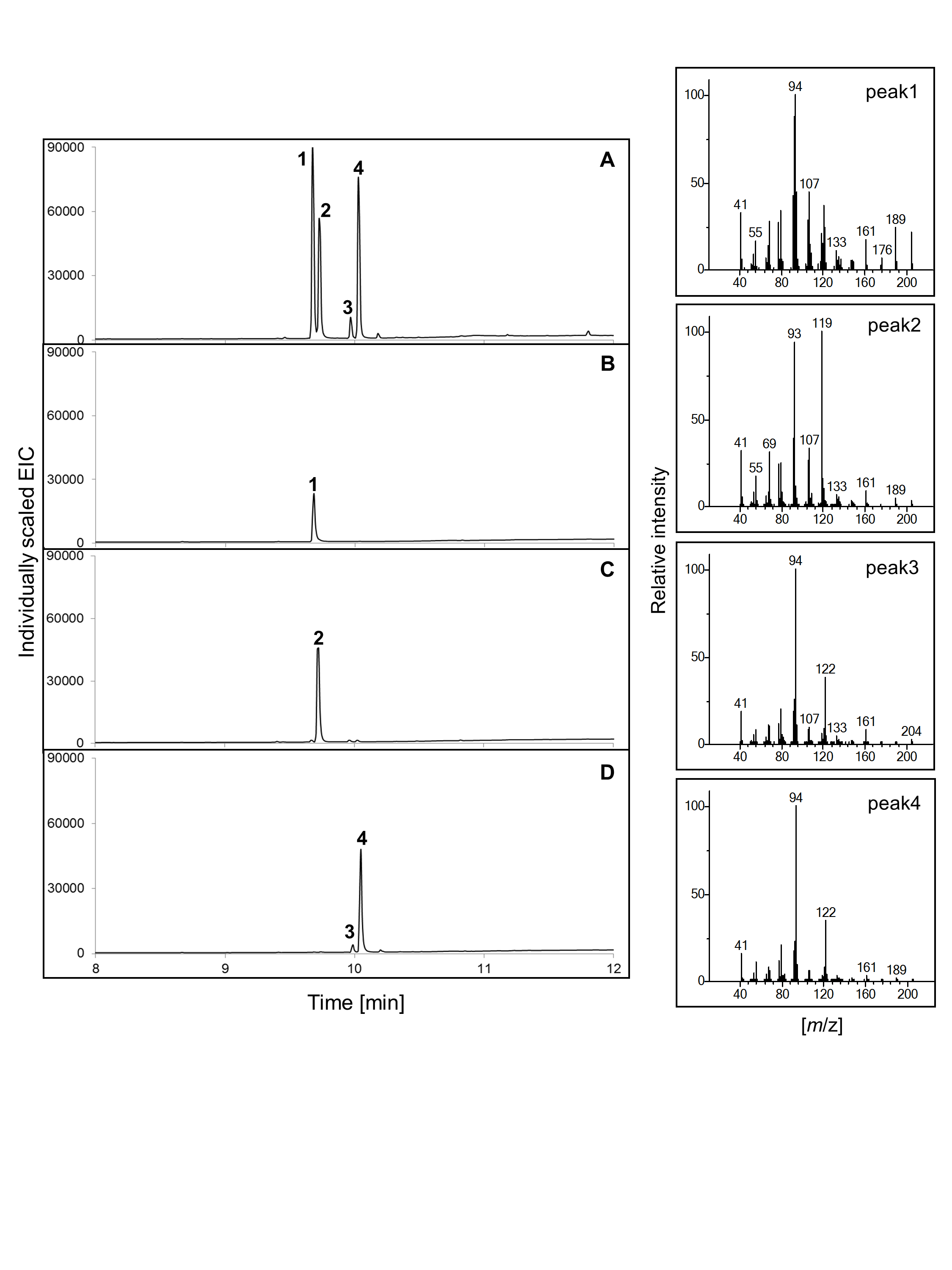

Supplement: Figure S3 — GCMS analysis (extracted ion chromatogram) of a sesquiterpene mixture and fractions separated by TLC. The sesquiterpene mixture was produced with a recombinant yeast strain expressing SaSSy (10) and was provide to us by Allylix Inc. It contained (A) α-santalene (1), α-exo-bergamotene (2), epi-β-santalene (3), β-santalene (4). The mixture was separated by TLC into three fractions containing mainly (B) α-santalene (1); (C) α-exo-bergamotene (2); or (D) β-santalene (4). Mass spectra of peaks 1 to 4 are provided. Peak numbers match the numbers in Table 1 and Figure 1. (TIF) [file pone.0075053.s003.tif]

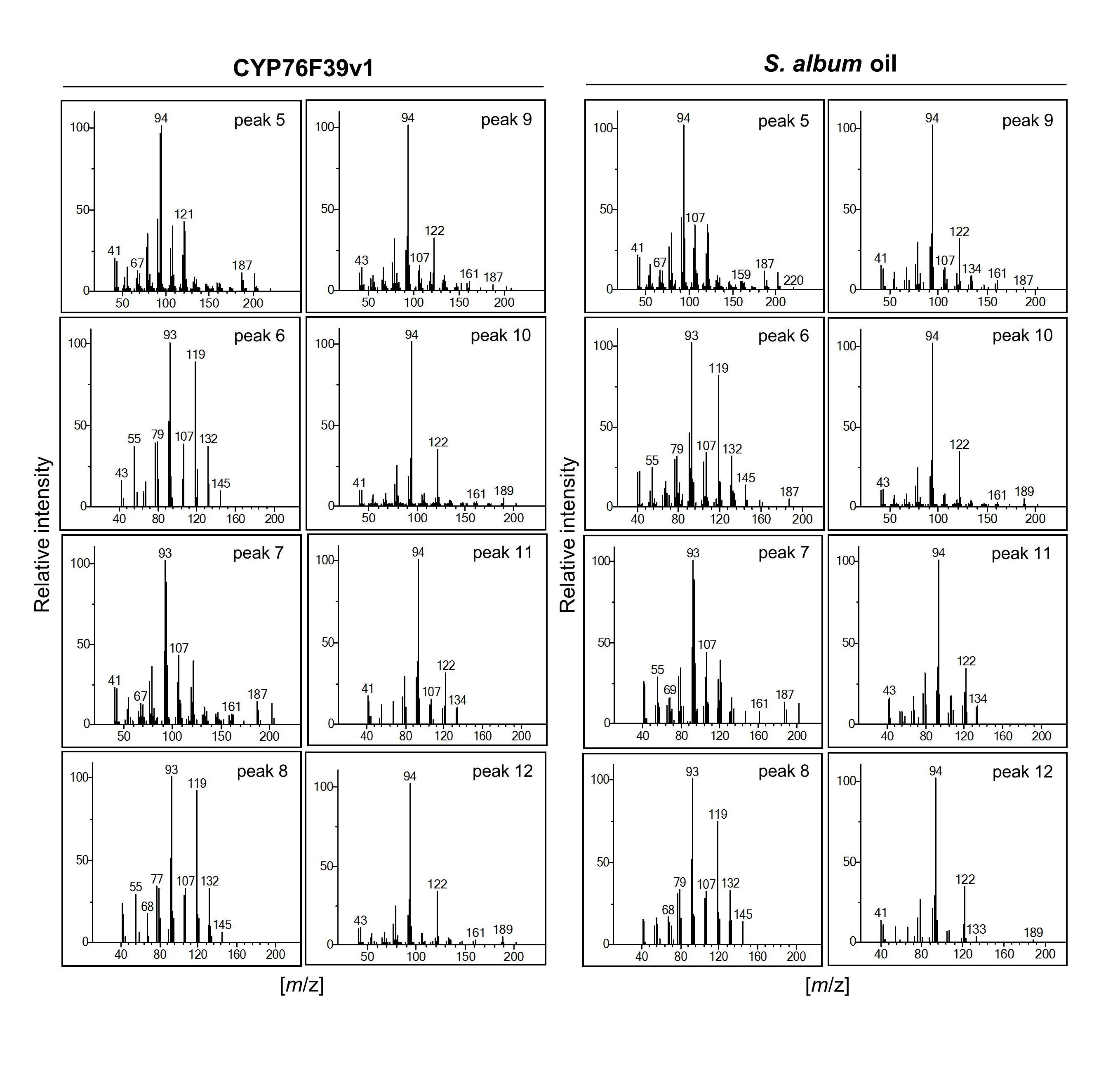

Supplement: Figure S4 — Mass spectra of products formed in vitro with Sa CYP76F39v1. Mass spectra of compounds corresponding to peaks 5 - 12 shown in Figure 3 and identified in assays with CYP76F39v1 (left panel) and S. album oil (right panel). Peak numbers match the numbers in Table 1, Figure 1, and Figure 3. (JPG) [file pone.0075053.s004.jpeg]

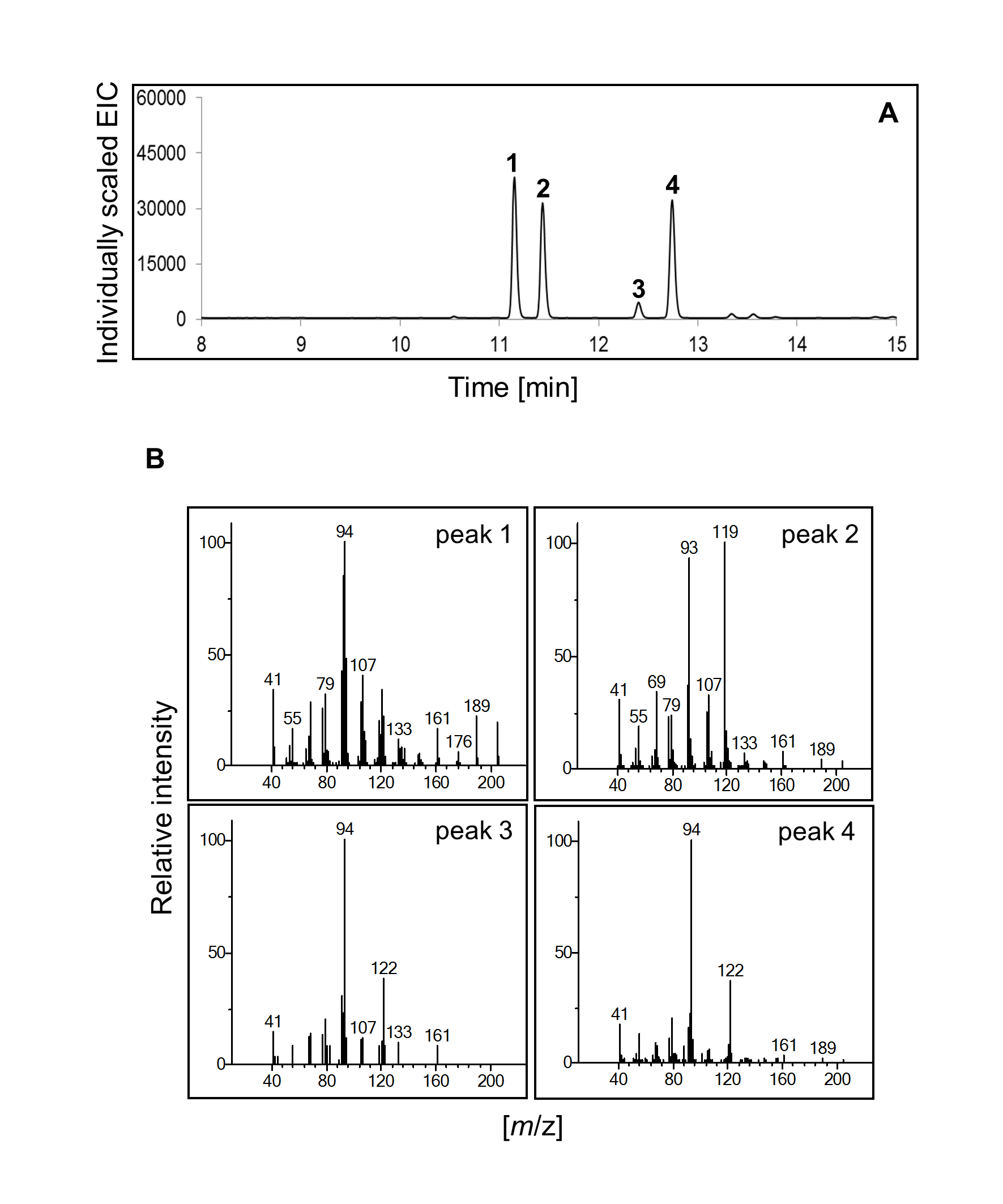

Supplement: Figure S6 — GCMS analysis (extracted ion chromatogram) and mass spectra of sesquiterpenes produced in yeast expressing Sa SSy. (A) GCMS analysis of sesquiterpenes extracted from pelleted yeast cells expressing SaSSy. (B) Mass spectra of peaks 1–4: α-santalene (1), α-exo-bergamotene (2), epi-β-santalene (3), and β-santalene (4). Compounds were identified by comparison to an authentic standard and retention indices. Peak numbers match the numbers in Table 1 and Figure 1. (TIF) [file pone.0075053.s006.tif]
