# Supplementary material for: Biosynthesis of Sandalwood Oil: Santalum album CYP76F Cytochromes P450 Produce Santalols and Bergamotol
Source: PLoS One. 2013 Sep 18;8(9):e75053. doi: 10.1371/journal.pone.0075053 (PMC3854609; doi:10.1371/journal.pone.0075053)
Supplement: Table S1 — Summary of transcriptome mining for CYP76 family members in the S. album Sanger and 454 sequence data. (DOCX) [file pone.0075053.s010.docx]

**Table S1**. Primers designed for amplification of cDNAs from *S. album*

| **Primer** | **Sequence (5′ → 3′)** | **Tm (°C)** |
| --- | --- | --- |
| Isogroup 1 Forward | ATGGACTTCTTAAGTTTTATCCTGTTTG | 55 |
| Isogroup 1 Reverse | TTACCCCCGGATCGGGACAG | 56 |
| Isogroup 2 Forward | ATGGACTTCTTAAGTTGTATCCTG | 52 |
| Isogroup 2 Reverse | TTACCCCCGGATTGGGACAG | 54 |
| SaCPR1 Forward | ATGAGTTCGAGCTCGGAGCTATG | 57 |
| SaCPR1 Reverse | TCACCACACATCCCGTAAATACCTTC | 57 |
| SaCPR2 Forward | ATGCAATTGAGCTCCGTCAAG | 58 |
| SaCPR2 Reverse | TCACCACACATCCCGTAAATACCTTCC | 58 |
