# Supplementary material for: Biosynthesis of Sandalwood Oil: Santalum album CYP76F Cytochromes P450 Produce Santalols and Bergamotol
Source: PLoS One. 2013 Sep 18;8(9):e75053. doi: 10.1371/journal.pone.0075053 (PMC3854609; doi:10.1371/journal.pone.0075053)
Supplement: Table S2 — Primers designed for amplification of cDNAs from S. album. (DOCX) [file pone.0075053.s011.docx]

**Table S2.** Summary of transcriptome mining for CYP76 family members in the *S. album* Sanger and 454 sequence data.

| Isogroup/  isotig | isotig | Reads | E-value  (CrCYP76B6 | Identity [%]  (CrCYP76B6) |
| --- | --- | --- | --- | --- |
| 1 | isotig05182  isotig05183  isotig05184 | 910  763  470 | 8.34E-142  2.68E-145  1.61E-78 | 71  71  52 |
| 2 | isotig06871  isotig06872 | 110  118 | 1.23E-126  9.19E-156 | 83  83 |
|  | isotig14788 | 11 | 1.53E-93 | 86 |
|  | isotig29133 | 1 | 1.49E-52 | 60 |

Note: Dotted lines denote isotig members of the same isogroup
